# Simple Meets Single: The Application of CRISPR/Cas9 in Haploid Embryonic Stem Cells

**DOI:** 10.1155/2017/2601746

**Published:** 2017-10-03

**Authors:** Zixi Yin, Lingyi Chen

**Affiliations:** State Key Laboratory of Medicinal Chemical Biology, Key Laboratory of Bioactive Materials, Ministry of Education, Collaborative Innovation Center for Biotherapy, Tianjin Key Laboratory of Protein Sciences, 2011 Collaborative Innovation Center of Tianjin for Medical Epigenetics and College of Life Sciences, Nankai University, Tianjin 300071, China

## Abstract

The CRISPR/Cas9 system provides a powerful method for the genetic manipulation of the mammalian genome, allowing knockout of individual genes as well as the generation of genome-wide knockout cell libraries for genetic screening. However, the diploid status of most mammalian cells restricts the application of CRISPR/Cas9 in genetic screening. Mammalian haploid embryonic stem cells (haESCs) have only one set of chromosomes per cell, avoiding the issue of heterozygous recessive mutations in diploid cells. Thus, the combination of haESCs and CRISPR/Cas9 facilitates the generation of genome-wide knockout cell libraries for genetic screening. Here, we review recent progress in CRISPR/Cas9 and haPSCs and discuss their applications in genetic screening.

## 1. Genome Editing

Genome editing refers to the techniques which allow desired modification of genomic DNA sequence. It is obvious that genome editing is a powerful tool for biomedical research, as well as for gene therapy. Thus, a genome editing tool with high editing efficiency, high DNA sequence specificity, and no unwanted byproducts is in great need.

Early genome editing techniques rely on the cellular endogenous homologous recombination. A donor DNA, containing a desired modified DNA fragment flanked by two long (several kilobase pairs) homologous arms, is introduced into living cells. Spontaneous homologous recombination mediated by the homologous arms occurs at extremely low frequency, ranging from 1 out of 10^3^ to 10^9^ cells [1, 2]. The spontaneous homologous recombination-based genome editing has been applied in genetic modifications of mouse embryonic stem cells, despite its low efficiency [3].

With the development of artificial site-specific nucleases, including zinc finger nucleases (ZFNs) [4, 5], transcription activator-like effector nucleases (TALENs) [6–10], and the clustered regularly interspaced short palindromic repeats (CRISPR)/CRISPR-associated protein (Cas) system [11, 12], the rate of homologous recombination is significantly elevated by producing a double-stranded break (DSB) at the desired site. This homology-directed repair (HDR) allows efficient homologous recombination-mediated genetic modifications in cells with a low homologous recombination rate, such as human embryonic stem cells and human induced pluripotent stem cells [10, 13]. In addition to HDR, DSBs can be repaired by nonhomologous end joining (NHEJ), which often generates deletion or insertion mutations. If a deletion or insertion mutation causes reading frame shift of a gene, the function of the gene is usually disrupted. Thus, artificial site-specific nucleases have been used to knock out genes through the NHEJ pathway [6, 7, 9].

ZFNs are the first generation of artificial site-specific endonucleases used for genome editing [4, 5]. ZFNs are fusion proteins composed of several zinc finger DNA-binding domains (ZFDBD) coupled to the FokI endonuclease catalytic domain. Each ZFDBD binds to a specific nucleotide triplet to recognize specific genetic loci. The binding specificity and the cutting efficiency of ZFDBD are relatively low. Therefore, the design and construction of ZFNs are difficult [14, 15].

The second generation of artificial site-specific endonucleases for genome editing is TALENs. Similar to ZFNs, TALENs consist of a customized DNA-binding domain and a FokI endonuclease catalytic domain. The DNA-binding domain is an array of tandem repeats. Each repeat has 33–35 amino acids. The amino acids in each repeat are nearly identical, except for amino acids 12 and 13. These two amino acids, which are known as repeat variable diresidue (RVD), determine the nucleotide binding specificity of an individual repeat [16, 17]. Thus, the number and the order of repeats in the DNA-binding domain specify its recognition sequence. In comparison with ZFNs, the design of TALENs is much easier and the specificity of TALENs is better. However, the cutting efficiency of TALENs is still low and variable at different loci. And assembly of the tandem repeats requires multiple rounds of molecular cloning.

The CRISPR/Cas9 system emerges as the third generation of artificial site-specific endonucleases for genome editing [11, 12]. The CRISPR/Cas9 system from *Streptococcus pyogenes* is a ribonucleoprotein complex composed of the RNA-guided Cas9 nuclease, noncoding CRISPR RNA (crRNA), and trans-activating CRISPR RNA (tracrRNA). crRNA directs the Cas9 complex to the target DNA site complementary to a short RNA guide, and then, Cas9 produces site-specific DSBs [18]. crRNA and tracrRNA fusion transcripts, also named single-chain guide RNAs (sgRNAs), are able to direct Cas9 to the target DNA sites, rendering the system simpler [11, 12]. Therefore, the two-component CRISPR/Cas9 system with Cas9 and sgRNA is most widely used. By changing the guide sequence of sgRNA, Cas9 can cut the genomic DNA with GN_20_GG motifs [19]. There are at least two advantages of CRISPR/Cas9, compared to TALENs. First, CRISPR/Cas9 is generally more efficient than TALENs [11, 12, 20]. Second, the construction of the sgRNA vector is more convenient than assembling the tandem repeats of TALENs. Thus, the CRISPR/Cas9 system opens a new era for genome editing. However, there are still some concerns regarding the off-target effect of CRISPR/Cas9 [21, 22]. Using a Cas9 nickase mutant and paired sgRNAs to produce DSBs reduces the off-target risk [23]. Alternatively, the specificity of Cas9 can be improved by a point mutation of Cas9 to optimize the contacts between Cas9 and DNA [24, 25]. More comprehensive introduction of the CRISPR/Cas9 system can be found in other reviews [26, 27].

## 2. Genetic Screening with Genome-Edited Haploid Embryonic Stem Cells

Pluripotent stem cells (PSCs), including embryonic stem cells (ESCs) and induced pluripotent stem cells (iPSCs), are able to self-renew infinitely and maintain the developmental potential into all cell types in the body. Thus, PSCs are promising sources of donor cells in regenerative medicine. Before transplanting autologous cells derived from PSCs into patients, genome editing is required to correct the mutated genes [28, 29]. In addition, genetically modified animals can be derived from genetically modified PSCs, facilitating the study of *in vivo* gene functions. Moreover, genetically modified PSCs may be used as *in vitro* models to investigate gene functions in disease and development, as well as for drug screening. Thus, a precise and high-efficient genome editing technique will benefit the application of PSCs in basic research, drug discovery, and cell replacement therapy.

**Figure 1:**
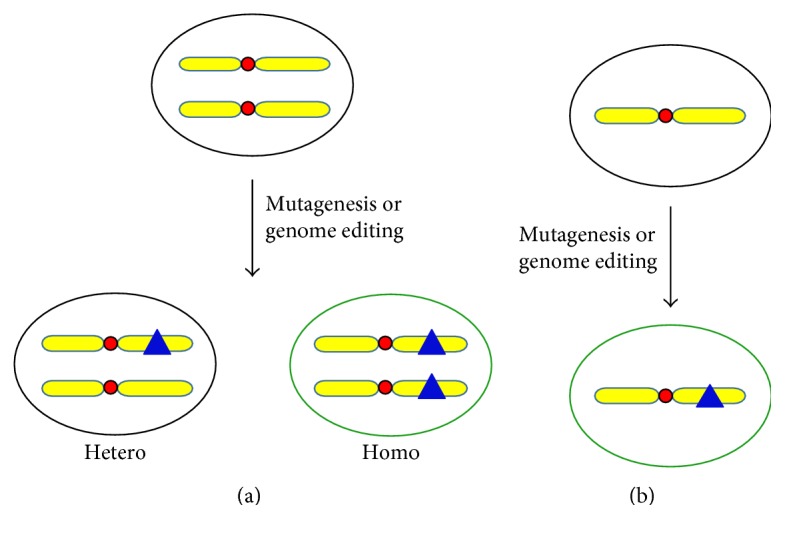
The advantage of haploid cells over diploid cells in genetic screening. (a) When a recessive mutation (shown by a blue triangle) is introduced into a diploid cell either by random mutagenesis or by genome editing, heterozygous or homozygous cells can be derived. However, the phenotype (illustrated by green circles) of the recessive mutation can be only detected in homozygous cells, but not in heterozygous cells. (b) Haploid cells only have one set of chromosomes. Once the recessive mutation is introduced into a haploid cell, the cell will display the corresponding phenotype.

Given the high efficiency and convenience of the CRISPR/Cas9 system, it has been applied not only in editing the individual gene in cells and organisms [20, 30, 31] but also in genome-wide knockout screening [32–34]. It is notable that human haploid cells were used in two CRISPR/Cas9-mediated knockout screening [32, 33]. Even though it is also doable to perform CRISPR/Cas9-mediated knockout screening in diploid cells [34], using haploid cells has obvious advantages over using diploid cells. In diploid cells, CRISPR/Cas9-mediated knockout may produce heterozygous and homozygous knockout cells. Heterozygous knockout cells may show no phenotype, leading to false-negative call. In contrast, each haploid cell only has one copy of each gene. Disruption of a gene will completely abolish the function of the gene and may reveal a certain phenotype if the gene has a function in the particular screening using haploid cells (Figure 1).

As described above, haploid PSCs (haPSCs) combined with the CRISPR/Cas9 system may provide a powerful genetic screening platform. However, it has been difficult to establish haPSCs. Frog haploid ESCs (haESCs) were first derived in 1970 [35]. Thirty-nine years later, the second haESCs from medaka fish were generated [36]. The first mammalian haESCs were generated from parthenogenetic mouse embryos produced by the artificial activation of oocytes, and fluorescence-activated cell sorting (FACS) is applied to select for haploid cells during continuous culturing [37, 38]. Subsequently, androgenetic mouse haploid ESCs were generated from androgenetic embryos produced by the removal of the maternal pronucleus or by sperm injection into enucleated oocytes [39, 40]. Mammalian haESCs from other species, including rat, monkey, and human, have also been established [41–44].

With the emergence of haESCs, genetic screenings have been carried out in these cells with only one set of chromosomes (summarized in Table 1). Mutated mouse haESC libraries have been generated by gene trap piggyBac transposon or retrovirus [37, 38, 45]. Using these cells, *Msh2* and *Hprt* have been identified as mismatch repair genes [37]. In another screening for genes involved in ricin toxicity, disruption of the GPCR Gpr107, as well as genes in the fucosylation pathway, renders these mutated cells resistant to ricin [38]. haESCs have also been applied to screen for genes required for the exit from self-renewal, and novel differentiation factors including Zfp706 and Pum1 were discovered [45]. These screenings have demonstrated the advantages of using haESCs for genetic screening. Recently, chemically mutagenized haESC libraries have been used for identifying genes mediating 6-thioguanine (6-TG) toxicity. Exome sequencing of individual 6-TG-resistant clones is required to identify the mutated genes [46]. Thus, it might be a hurdle for the application of chemically mutagenized haESC libraries in genetic screenings.

In above-mentioned screenings, mutations were introduced into haESCs by insertion of transposon- or retrovirus-mediated gene trap cassettes, except for chemical mutagenesis. The gene trap cassette has to be integrated downstream of an active promoter to disrupt a gene, resulting in low efficiency of mutagenesis. For example, only less than 1000 mutated colonies can be selected out, after transfection of 1 *μ*g transposase plasmid and 1–40 *μ*g gene trap cassette containing a donor vector into 5 × 10^6^ diploid ESCs [47]. Therefore, to generate a gene-trapped cell library covering the whole genome, a large number of starting cells are required. Moreover, only actively transcribed genes can be trapped, and inactive genes are likely not mutated. In contrast, if CRISPR/Cas9 is used for mutagenesis to generate a mutated cell library, a mutation will be introduced as long as Cas9 and sgRNA is coexpressed in a cell, regardless of the integration site of the sgRNA vector or the transcription status of the gene. Thus, a mutated cell library produced by CRISPR/Cas9 has a better coverage of the genome. In addition, using focused sgRNA plasmid libraries, one can select a specific set of genes to be mutated and construct a customized mutated cell library. For example, a lentiviral paired-guide RNA (pgRNA) library targeting long noncoding RNAs (lncRNAs) has been constructed and applied in establishing a lncRNA knockout library of human cancer cells [48]. Another advantage of using CRISPR/Cas9 for mutagenesis is that the mutated gene can be easily identified by analyzing the integrated sgRNA vector. Combined with a high-throughput sequencing technique, genetic screenings for certain quantitative traits, such as growth rate, are plausible [48].

**Figure 2:**
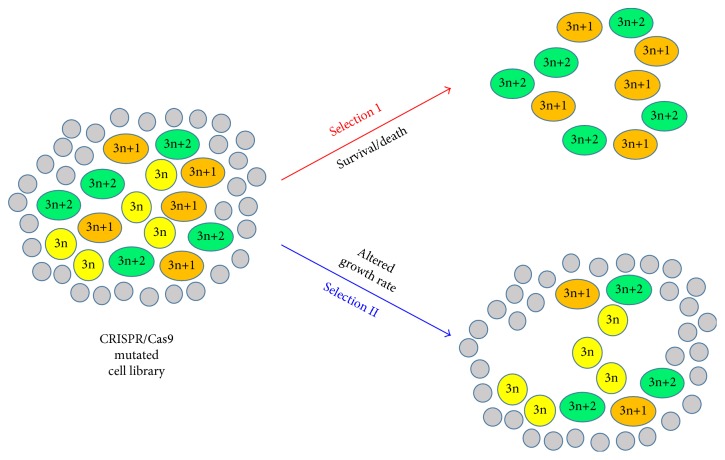
The limitation of CRISPR/Cas9 in genetic screening. Each circle or oval represents a cell. 3n and 3n+1 and 3n+2 in yellow circles and orange and green ovals, respectively, represent the different types of InDels caused by a specific sgRNA. Grey circles are cells harboring other sgRNAs, but not the specific sgRNA mentioned above. In the first genetic screening (selection I), the disruption of the gene targeted by the specific sgRNA allows cell survival. Thus, only cells with 3n+1 and 3n+2 InDels, but not cells with 3n InDels, will survive after the selection. The integrated sgRNA sequence can be easily identified from the surviving cells. In the second genetic screening (selection II), the disruption of the gene targeted by the specific sgRNA renders cells to grow slower. To identify sgRNA causing the slow growth of the phenotype, high-throughput sequencing of the integrated sgRNA sequence and quantification of the relative amount of individual sgRNAs are required. However, cells with 3n InDels and an unchanged growth rate will interfere with the quantification, because cells with 3n, 3n+1, and 3n+2 InDels have the same sgRNA. Therefore, the observed reduction of cells with the specific sgRNA (including 3n, 3n+1, and 3n+2) is less than the actual reduction of cells with a disruption mutation (3n+1 and 3n+2). Therefore, the 3n mutation might lead to a false-negative hit.

Despite the advantages mentioned above, CRISPR/Cas9 has its own limitation when used for the construction of mutated cell libraries. Each sgRNA might generate three types of insertion or deletion (InDel) mutations, 3n, 3n+1, and 3n+2. Of these three mutations, 3n InDels lead to insertion or deletion of a few amino acids in proteins and might not disrupt the function of proteins. Even though cells with 3n InDels are functionally intact, they are categorized as mutated cells when analyzing the integration of the sgRNA vector. In another word, about 1/3 of cells in a CRISPR/Cas9 mutated cell library are not authentic mutated cells. These cells might not interfere with genetic screenings in which only mutated cells survive after the selection. Nevertheless, for genetic screenings by comparing cell populations before and after selection through deep sequencing, these cells would be background noise and result in false-negative hits (Figure 2).

## 3. Genetic Screening in Mice Derived from haESCs

haPSCs not only provide a unique platform for genetic screening but also allow the generation of genetically modified semicloned mice. Live mice with a low birth rate (4.5%) can be obtained by injection of androgenetic haPSCs into MII oocytes [39, 40]. Similarly, cytoplasmic injection of parthenogenetic haPSCs into androgenetic embryos produces live mice [49]. The low birth rate of the so called semicloned mice is likely due to aberrant epigenetic status of imprinted genes in haPSCs. The removal of differentially methylated regions (DMRs) controlling two paternally repressed imprinted genes, *H19* and *Gtl2*, from haESCs improves the birth rate of semicloned mice to 15–20% [50, 51]. When genetically modified haESCs are injected into mature oocytes, the resulting semicloned mice are heterozygous [51]. These heterozygous semicloned mice may not be suitable for genetic screening. Yet, they have a great application value in studying diseases caused by multiple heterozygous gene mutations, because of the high efficiency of targeted mutagenesis by CRISPR/Cas9 in haESCs. Alternatively, semicloned mice with biallelic mutations may be generated by injecting haESCs stably expressing Cas9 and sgRNA into oocytes [51]. Even though the birth rate of semicloned mice has been increased to 20%, the generation of semicloned mice is still relatively labor intensive. Moreover, a large-scale genetic screening with a knockout mouse library is cost expensive and requires a lot of space. Thus, it is more feasible to perform small-scale genetic screening with semicloned homozygous mutant mice.

## 4. Conclusions and Future Directions

haESCs have only one set of chromosomes in each cell, hence providing an excellent platform for genetic screening. In the past few years, the advantages of haESCs in genetic screening have been demonstrated. To make haESCs more convenient for genetic screening, one issue has to be solved. Due to spontaneous diploidization, haESCs has to be sorted every few passages to select for haploid cells. Efforts have been made to prevent the diploidization of haESCs. Inhibition of MEK and GSK3 by the inhibitors PD0325901 and CHIR99021 (2i) are critical for the stabilization of the haploid karyotype [37–40]. Using the Wee1 kinase inhibitor PD166284 to accelerate the G2/M phase transition also reduces the rate of spontaneous diploidization, allowing continuous culturing of haESCs for 4 weeks without the need for fluorescence-activated cell sorting [52]. Establishment of haESCs with a stable haploid karyotype will promote the application of haESCs for genetic screening.

The CRISPR/Cas9 system allows efficient targeted mutagenesis, facilitating the generation of a genome-wide knockout haESC library. Yet, to our best knowledge, genetic screening using haESCs and CRISPR/Cas9 has not been reported. In the near future, it is expected that various genetic screenings based on haESCs and CRISPR/Cas9 will be carried out, facilitating the discoveries and advancement in biological science.

## Figures and Tables

**Table 1:** Genetic screens using mouse parthenogenetic haESCs.

Mutagenesis method	Screening strategy	Key factors identified	Reference
PiggyBac transposon	Resistance to 2-amino-6-mercaptopurine to screen for genes involved in DNA mismatch repair	*Msh2* and *Hprt*	[37]
Retroviral gene trap	Resistance to ricin to screen for genes mediating ricin toxicity	*Gpr107* and genes in the fucosylation pathway	[38]
PiggyBac transposon	Resistance to 6-thioguanine to screen for genes involved in DNA mismatch repair	*Msh2*, *Msh6*, and *Mlh1*	[53]
	Resistance to olaparib to screen for genes mediating olaparib toxicity	*Parp1*	
PiggyBac transposon	Using a *Rex1-GFP* reporter to screen for genes required for the exit from self-renewal	*Zfp706* and *Pum1*	[45]
Retroviral gene trap	Using an X-linked inducible Xist gene to screen for genes required for X chromosome inactivation	*Spen*	[54]
Ethyl methanesulfonate	Resistance to 6-thioguanine to screen for genes involved in DNA mismatch repair	*Hprt*, *Dnmt1*, and genes for DNA mismatch repair	[46]
